# Hydroalcoholic Extracts of *Cucumis prophetarum* L. Affect the Insulin Signaling Pathway in an In Vitro Model of Insulin-Resistant L6 Myotubes

**DOI:** 10.3390/molecules31020307

**Published:** 2026-01-15

**Authors:** Zewdie Mekonnen, Giuseppe Petito, Getasew Shitaye, Gianluca D’Abrosca, Belete Adefris Legesse, Sisay Addisu, Antonia Lanni, Roberto Fattorusso, Carla Isernia, Lara Comune, Simona Piccolella, Severina Pacifico, Rosalba Senese, Gaetano Malgieri, Solomon Tebeje Gizaw

**Affiliations:** 1 Department of Biochemistry, School of Medicine, College of Health Sciences, Addis Ababa University, Addis Ababa P.O. Box 9086, Ethiopia; zewdie.mekonnen@aau.edu.et (Z.M.); sisay.addisu@aau.edu.et (S.A.); 2 Department of Biomedical Sciences, College of Medicine and Health Sciences, Bahir Dar University, Bahir Dar P.O. Box 79, Ethiopia; getasewshitaye.ayalew@unicampania.it; 3 Department of Environmental, Biological, and Pharmaceutical Sciences and Technologies, University of Campania, 81100 Caserta, Italy; giuseppe.petito@unicampania.it (G.P.); antonia.lanni@unicampania.it (A.L.); roberto.fattorusso@unicampania.it (R.F.); carla.isernia@unicampania.it (C.I.); lara.comune@unicampania.it (L.C.); simona.piccolella@unicampania.it (S.P.); severina.pacifico@unicampania.it (S.P.); rosalba.senese@unicampania.it (R.S.); 4 Department of Human Sciences, Link Campus University, Via del Casale di S. Pio V, 44, 00165 Roma, Italy; 5 Center for Innovative Drug Development and Therapeutic Trials for Africa (CDT-Africa), College of Health Sciences, Addis Ababa University, Addis Ababa P.O. Box 9086, Ethiopia; belete.adefris@aau.edu.et

**Keywords:** type 2 diabetes mellitus, *Cucumis prophetarum* L., insulin resistance, apigenin-*C*-glycosides, cucurbitacins

## Abstract

Type 2 diabetes mellitus (T2DM) can be traditionally treated by edible and medicinal species rich in flavonoids and triterpenoids known for their metabolic benefits. *Cucumis prophetarum* L. has shown antioxidant and antidiabetic properties in decoction extracts. Since solvent polarity strongly influences the extraction of secondary metabolites, this study investigated the hydroalcoholic extracts of *C. prophetarum* L. to explore their chemical composition and insulin-sensitizing potential. Hydroalcoholic extracts from the leaf, stem, and root of *C. prophetarum* L. were analyzed by UV-Vis spectroscopy, ATR-FTIR, and UHPLC-ESI-QqTOF–MS/MS to profile their secondary metabolites. The insulin-sensitizing potential of each extract was assessed using an in vitro model of palmitic-acid-induced insulin resistance in L6 skeletal muscle cells, followed by Western blot analysis of key insulin-signaling proteins. Flavonoid glycosides such as apigenin-*C*,*O*-dihexoside, apigenin-malonylhexoside, and luteolin-*C*,*O*-dihexoside were abundant in leaf and stem extracts, while cucurbitacins predominated in the root. MTT assay confirmed that hydroalcoholic stem and root extracts of *C. prophetarum* L. were non-cytotoxic to L6 myotubes, whereas the leaf extract reduced viability only at higher concentrations. Oil Red O staining revealed a pronounced decrease in lipid accumulation following stem and root extract treatment. Consistently, the stem extract enhanced insulin signaling through the activation of the IRS-1/PI3K/Akt pathway, while the root extract primarily modulated the AMPK–mTOR pathway. Importantly, both extracts promoted GLUT4 translocation to the plasma membrane, highlighting their complementary mechanisms in restoring insulin sensitivity. Hydroalcoholic extracts of *C. prophetarum* L. alleviate insulin resistance through multiple molecular mechanisms, with bioactivity and composition differing markedly from previously reported in the decoctions, which highlight a promising source of insulin-sensitizing phytochemicals and underscore the importance of solvent selection in maximizing therapeutic potential.

## 1. Introduction

The therapeutic use of medicinal plants as foundations for modern drug development has deep historical roots, particularly for managing chronic and infectious diseases that continue to re-emerge. Their widespread availability, low cost, and richness in bioactive metabolites make them valuable resources in both traditional and modern medicine alike [1]. Among the plant families of notable medicinal and nutritional importance is Cucurbitaceae [2], well known for its cucurbitacin content, bitter triterpenoid compounds predominantly found in the roots [3]. Edible fruits from this family, such as pumpkin, zucchini, watermelon, melon, and cucumber, are widely consumed and appreciated not only for their nutritional value but also for their diverse biological properties, including antifungal, antibacterial, antiviral, antidiabetic, and anticancer activities [4,5,6,7]. Within this family, *Cucumis prophetarum* (commonly known as wild gourd or wild cucumber) has gained increasing attention for its medicinal potential. The species is widely distributed across Africa (including Ethiopia), Asia, and Australia [8,9,10,11]. Beyond its traditional use in treating various ailments, recent investigations have demonstrated the antidiabetic and antioxidant potential of its fruit extracts obtained using different solvents [12]. Phytochemical screening of the aqueous leaf extract and methanol fruit extract of the *C. prophetarum* was reported to possess tannins, steroids, flavonoids, triterpenoids, phenols, and saponins [13,14]. The aqueous fruit extract of the *C. prophetarum* from India was indicated to contain a novel N-trisaccharide (containing two hexoses and one inositol) that has antidiabetic properties [8]. In addition, decoction extract of *C. prophetarum* (leaf and stems mainly rich in apigenin *C*-glycosides, and the root decoction rich in raffinose and cucumegastigmane II) enhanced insulin sensitivity in insulin-resistant L6 myotube, suggesting a direct mechanism relevant to glucose metabolism [15]. However, the extraction method plays a crucial role in determining the qualitative and quantitative profile of bioactive metabolites, as hydroalcoholic and aqueous extractions often determine different chemical compositions [16,17].

Type 2 diabetes mellitus (T2DM) is a chronic metabolic disorder characterized by insufficient insulin production or ineffective insulin utilization, leading to impaired glucose uptake and persistent hyperglycemia [18,19]. To compensate for insulin resistance, peripheral tissues may signal the pancreas to secrete more insulin, resulting in hyperinsulinemia [20]. Its pathophysiology involves peripheral insulin resistance, dysregulated hepatic glucose production, and progressive β-cell dysfunction, ultimately leading to β-cell destruction and the consequent loss of insulin secretory capacity [21,22].

Insulin sensitivity in skeletal muscle is primarily regulated through the IRS-1/PI3K/Akt pathway, which mediates glucose uptake and glycogen synthesis, whereas the AMPK–mTOR axis plays a pivotal role in cellular energy homeostasis and lipid metabolism [23,24]. Dysregulation of either pathway contributes to the pathogenesis of T2DM, highlighting them as key targets for therapeutic intervention [25].

Despite the availability of several pharmacological agents, such as insulin sensitizers, α-glucosidase inhibitors, and drugs that enhance endogenous insulin release [26,27,28], their long-term efficacy is often limited by β-cell decline and mechanism-related adverse effects. These limitations have prompted the search for plant-derived alternatives capable of improving insulin sensitivity and glucose metabolism.

In this context, the present study aims to elucidate the biological effects of hydroalcoholic extracts derived from the leaf, stem, and root of *C. prophetarum* in an in vitro model of palmitate-induced insulin resistance in L6 myotubes, with particular emphasis on their ability to modulate key components of the insulin signaling cascade, including the IRS-1/PI3K/Akt and AMPK–mTOR pathways.

Furthermore, the extracts were subjected to comprehensive secondary metabolite profiling using UV spectrophotometry, Attenuated Total Reflection (ATR) Fourier-transform infrared spectroscopy (FTIR), and ultra-high-performance liquid chromatography coupled with high-resolution tandem mass spectrometry (UHPLC-HR-MS/MS).

## 2. Results

### 2.1. Spectroscopic Prediction of Hydroalcoholic Extracts

**Figure 1:**
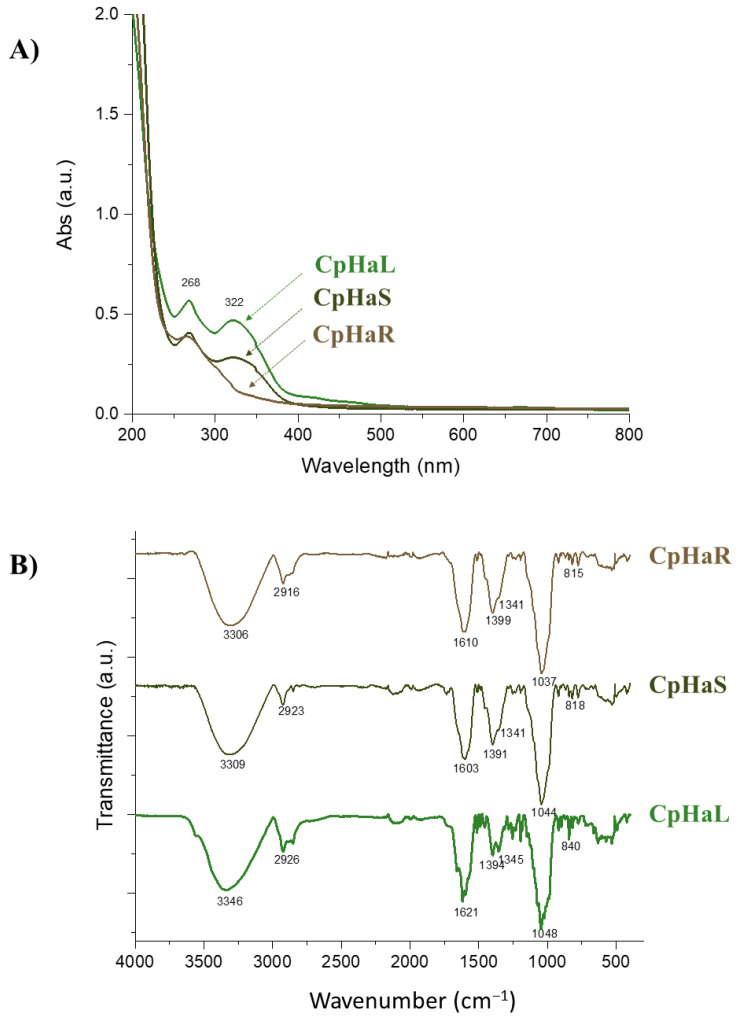
Spectroscopic data of the hydroalcoholic extracts of *C. prophetarum* leaf (CpHaL), stem (CpHaS) and root (CpHaR). (**A**): UV-Vis spectra, (**B**) ATR-FT-IR spectra.

The hydroalcoholic solution exhausted the *C. prophetarum* leaf, stem, and root with a yield of 5.99%, 3.32%, and 1.81%, respectively. Upon the UV-Vis spectral analysis of the hydroalcoholic extracts, two prominent absorption bands at 268 nm and 322 nm in both leaf and stem extracts were observed (Figure 1A).

These bands are characteristic of the flavone chromophore, suggesting the presence of conjugated aromatic systems. Specifically, the band at 268 nm corresponds to Band II of flavones, associated with π–π* transitions in the benzoyl moiety (typically 240–280 nm), while the band at 322 nm is indicative of band I, attributed to the cinnamoyl system (typically 300–380 nm). These features were markedly less evident in the root extract, suggesting a lower abundance of flavonoid-type compounds.

The ATR-FT-IR spectra provided further insight into the functional groups present in the extracts (Figure 1B). A broad band between 3100 and 3600 cm^−1^ indicated O–H stretching vibrations and hydrogen bonding, likely due to hydroxylated phenolics such as flavonoids. Absorptions within the 2750–3100 cm^−1^ region were attributed to aliphatic C–H stretching vibrations, potentially arising from CH_2_ and CH_3_ groups. Notably, the root extract exhibited distinct features in this region that may correspond to asymmetric and symmetric C(sp^3^)–H stretching of methylene groups found in tetracyclic triterpenes, such as cucurbitacins [11,29,30]. A sharp peak at 2923 cm^−1^ may be linked to the methylene C–H stretching of flavone *C*-glycosides, compounds previously reported in other *Cucumis* species [31]. In the carbonyl region (1750–1500 cm^−1^), bands at 1621 cm^−1^ (leaf) and 1603 cm^−1^ (stem) suggest C=O stretching of acetylated flavonoids [32,33], whereas the strong absorption at 1610 cm^−1^ in the root extract may reflect ketonic carbonyl groups typical of cucurbitacins. Additional C=C aromatic stretching signals, observed between 1445 and 1250 cm^−1^, further support the occurrence of flavonoids in leaf and stem extracts. Finally, characteristic C–O stretching vibrations due to glycosidic bonds were detected between 1100 and 1030 cm^−1^, with defined bands at 1048, 1044, and 1037 cm^−1^ [34].

### 2.2. UHPLC-ESI-QqTOF-HR-MS/MS Analyses Ascertain the Existence of Diversified Compounds in the C. prophetarum Hydroalcoholic Extracts

**Figure 2:**
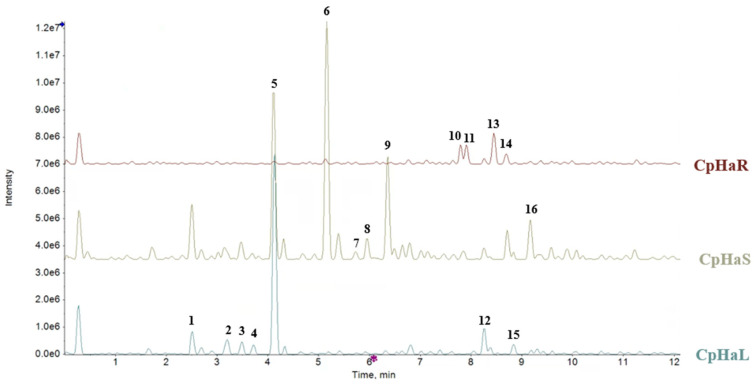
Total Ion Chromatograms (TIC) of hydroalcoholic extracts from *Cucumis prophetarum* leaf (CpHaL), stem (CpHaS), and root (CpHaR) acquired using UHPLC-QqTOF-MS in negative ion mode with electrospray ionization (ESI).

UHPLC-ESI-QqTOF HR-MS/MS analyses were performed to deeply understand the chemical composition of the hydroalcoholic extracts of *C. prophetarum*. The Total Ion Current (TIC) chromatograms and corresponding TOF-MS data reporting the experimental masses, retention times, molecular formulas, and [M−H]^−^ ions of the detected compounds are presented in Figure 2 and Table 1. TOF-MS/MS spectra of tentatively assigned compounds are available as Supplementary Materials (Supplementary Figures).

Consistent with UV spectroscopic findings, glycosylated flavones were predominantly found in the leaf and stem extracts. Among them, apigenin-8-*C*-hexoside-7-*O*-hexoside (apigenin-*C*,*O*-dihexoside) (**5**) was particularly abundant in the leaf extract, while the stem extract also contained apigenin-8-*C*-hexoside (**6**) (Supplementary Figures S1 and S2). Compound **5** exhibited a [M−H]^−^ ion at *m*/*z* 593.1515, and its MS/MS spectrum showed fragment ions at *m*/*z* 473.1101 (due to a 120.04 Da loss via cross-ring cleavage of the *C*-linked hexose) and *m*/*z* 431.096 (corresponding to the loss of a dehydrated hexose). Further fragmentation led to ions at *m*/*z* 341.0667 (−90.03 Da) and 311.0552 (−120.05 Da), consistent with successive cross-ring cleavages [35]. Similar fragmentation patterns were observed for compound **6** ([M−H]^−^ at *m*/*z* 431.0985), which produced ions at *m*/*z* 283.0595 and 269.0461, the latter likely corresponding to the aglycone. The relative abundance of *m*/*z* 311.05 over 341.07 supports C-8 glycosylation. Isomeric compounds **7** and **8,** showing a neutral loss of 60.02 Da, were consistent with apigenin acetylhexosides differing by the site of acetylation. Compound **9** ([M−H]^−^ at *m*/*z* 517.0999) produced a fragment ion at *m*/*z* 473.1107 due to CO_2_ elimination, indicative of apigenin malonylhexoside [35]. Luteolin *C*,*O*-dihexoside (**3**) was also identified in both stem and leaf extracts, following fragmentation behavior similar to compound **5**. Hydroxycinnamoyl derivatives **2** and **4** were identified as regaloside A and regaloside F, respectively. Their [M−H]^−^ ions underwent a 236.09 Da neutral loss, likely corresponding to a dehydrated hexosylglycerol moiety, leading to fragment ions at *m*/*z* 163.0401 (*p*-coumarate, compound **2**) and *m*/*z* 193.0505 (ferulate, compound **4**). These compounds were previously isolated from fresh bulbs of *Lilium regale* [35]. Additionally, benzyl pentosyl hexoside (compound **1**, [M−H]^−^ at *m*/*z* 401.1451) was detected in both leaf and stem extracts.

The root extract was characterized by the presence of cucurbitacins, triterpenoids typical of Cucurbitaceae species known for their defensive role against herbivores. Compound **10** ([M−H]^−^ at *m*/*z* 533.3128) was identified as cucurbitacin H. Its TOF-MS/MS spectrum showed a major fragment at *m*/*z* 479.2828, resulting from the sequential loss of three water molecules. Compounds **11** ([M−H]^−^ at *m*/*z* 515.3006) and **13** ([M−H]^−^ at *m*/*z* 517.3176) were consistent with isocucurbitacin D and dihydroisocucurbitacin D, respectively, both previously isolated from *C. prophetarum* fruits [36]. Cucurbitacin A (**14**) was also identified with a [M−H]^−^ ion at *m*/*z* 561.3072 (Supplementary Figure S3). Lastly, polyunsaturated fatty acids were detected in all three extracts, including hydroperoxyoctadecatrienoic acid (**12**), trihydroxyoctadecenoic acid (**15**), and hydroperoxyoctadecatrienoic acid (**16**) [37].

### 2.3. Stem Hydroalcoholic Extract of C. prophetarum Improves Insulin Signaling in Insulin-Resistant L6 Myotubes

**Figure 3:**
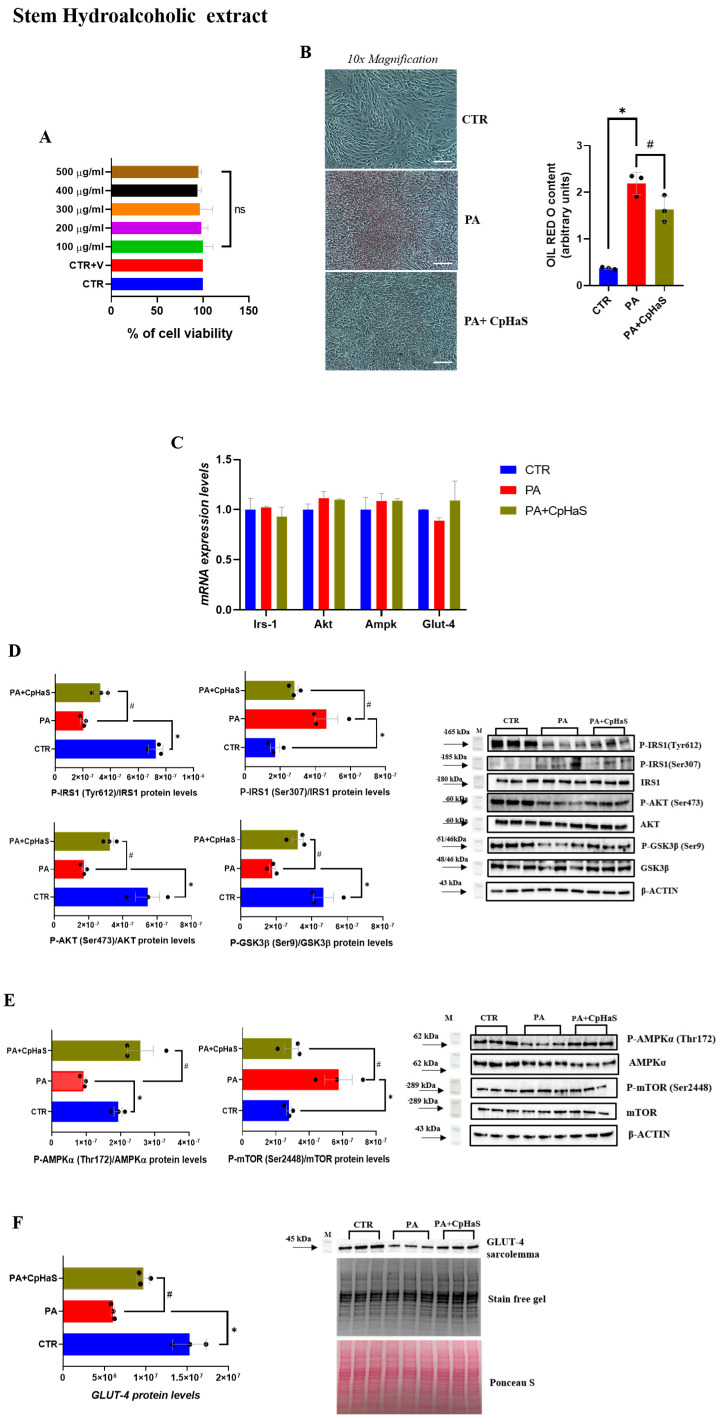
(**A**) MTT assay in L6 cells following the exposure to various concentrations of Stem Hydroalcoholic extract from *C. prophetarum* L. for 24 h; (**B**) ORO staining and ORO staining quantification normalized for protein content of L6 cells treated with PA (0.75 mM) and Stem Hydroalcoholic extract from *C. prophetarum* L. (300 µg/mL). Cells were visualized under a 10× magnification, with a scale bar of 2 μm; (**C**) mRNA expression levels of Irs-1, Akt, Ampk, Glut-4. The mRNA level was normalized to that of β-actin and GAPDH; (**D**,**E**) Representative immunoblots of P-IRS1 (Tyr612), P-IRS1 (Ser307), P-AKT (Ser473), P-GSK3β (Ser9), P-AMPKα (Thr172), and P-mTOR (Ser2448). Histograms represent the results of the densitometric analysis of the immunoblots, normalized to the corresponding total (non-phosphorylated) form of each protein and to β-actin as a loading control; (**F**) Representative immunoblots of GLUT4 detected in the plasma membrane fraction. Histograms represent the results of the densitometric analysis of the immunoblots. Stain-Free technology was used as normalization. Results are expressed as mean ± SEM (*n* = 3/group). One-way ANOVA was used for statistical analysis. * *p* < 0.05 vs. CTR; # *p* < 0.05 vs. PA. **CTR:** control; **PA:** palmitic acid; **PA + CpHaS:** palmitic acid plus stem hydroalcoholic extract.

Exposure of L6-myotubes to the stem hydroalcoholic extract of *C. prophetarum* (100–500 µg/mL) for 24 h did not significantly affect cell viability (Figure 3A). Oil Red O staining revealed a marked difference in lipid accumulation between PA-treated cells and the control group (Figure 3B). Notably, in the PA + CpHaS-treated group, a significant reduction in lipid accumulation was observed compared to PA-treated cells (Figure 3B). To investigate whether the stem hydroalcoholic extract exerts a protective effect against PA-induced defects in insulin signaling, the phosphorylation levels of key proteins involved in this pathway were analyzed by Western blotting at the selected concentration. Firstly, the expression levels of Irs-1, Akt, Ampk, and Glut-4 were evaluated and, as expected, were unchanged among the experimental groups (Figure 3C). Proteins belonging to the canonical insulin signaling pathway, including Insulin Receptor Substrate-1 (Tyr612 and Ser307), Akt (Ser473), and Glycogen Synthase Kinase-3 beta (Ser9), were evaluated. Moreover, proteins of the metabolic/energy-sensing pathway, such as AMP-activated Protein Kinase α (Thr172) and the Mechanistic Target of Rapamycin (Ser2448), were also assessed to investigate their role in insulin sensitivity and energy regulation.

The exposure of L6 myotubes to PA resulted in a significant reduction in IRS-1 phosphorylation at Tyr612 compared to the control group (Figure 3D). However, in the PA + CpHaS group, IRS-1 (Tyr612) phosphorylation levels were significantly increased compared to PA-treated myotubes (Figure 3D). To further characterize the insulin signaling pathway, IRS-1 phosphorylation at Ser307 was assessed. Phosphorylation at this site is widely recognized as a marker of insulin resistance [38]. Our results showed that Ser307 phosphorylation of IRS-1 was significantly increased in the PA group compared to the CTR group (Figure 3D). In contrast, treatment with PA + CpHaS significantly reduced IRS-1 Ser307 phosphorylation compared to PA-treated myotubes (Figure 3D). Consistent with the observed effects, we found that AKT phosphorylation was markedly decreased in PA-treated myotubes compared to the control group, whereas treatment with PA + CpHaS significantly restored AKT phosphorylation levels (Figure 3D). Moreover, GSK-3β phosphorylation at Ser9 was examined, revealing a decrease in PA-treated myotubes compared to the control group (Figure 3D). In contrast, GSK-3β phosphorylation was significantly increased in the PA + CpHaS-treated group compared to the PA group (Figure 3D).

Additionally, the effects of stem hydroalcoholic extract on AMPKα (Thr172) and mTOR (Ser2448) phosphorylation were evaluated. A significant decrease in AMPKα phosphorylation levels was observed in PA-treated myotubes compared to the CTR group, whereas treatment with PA + CpHaS significantly increased AMPKα phosphorylation compared with PA-treated myotubes (Figure 3E). Furthermore, our results showed a significant increase in mTOR (Ser2448) phosphorylation levels in PA group compared to the control group, while treatment with PA + CpHaS resulted in a marked decrease in mTOR phosphorylation (Figure 3E). Moreover, our results show that PA-treated L6 myotubes exhibit a marked reduction in GLUT4 membrane localization, thereby confirming the presence of an insulin-resistant state (Figure 3F). Furthermore, in the PA + CpHaS group, a significant enhancement of GLUT4 translocation to the plasma membrane was observed compared with PA-treated cells (Figure 3F).

### 2.4. Root Hydroalcoholic Extract of C. prophetarum Affects Insulin Signaling in Insulin-Resistant L6 Myotubes

**Figure 4:**
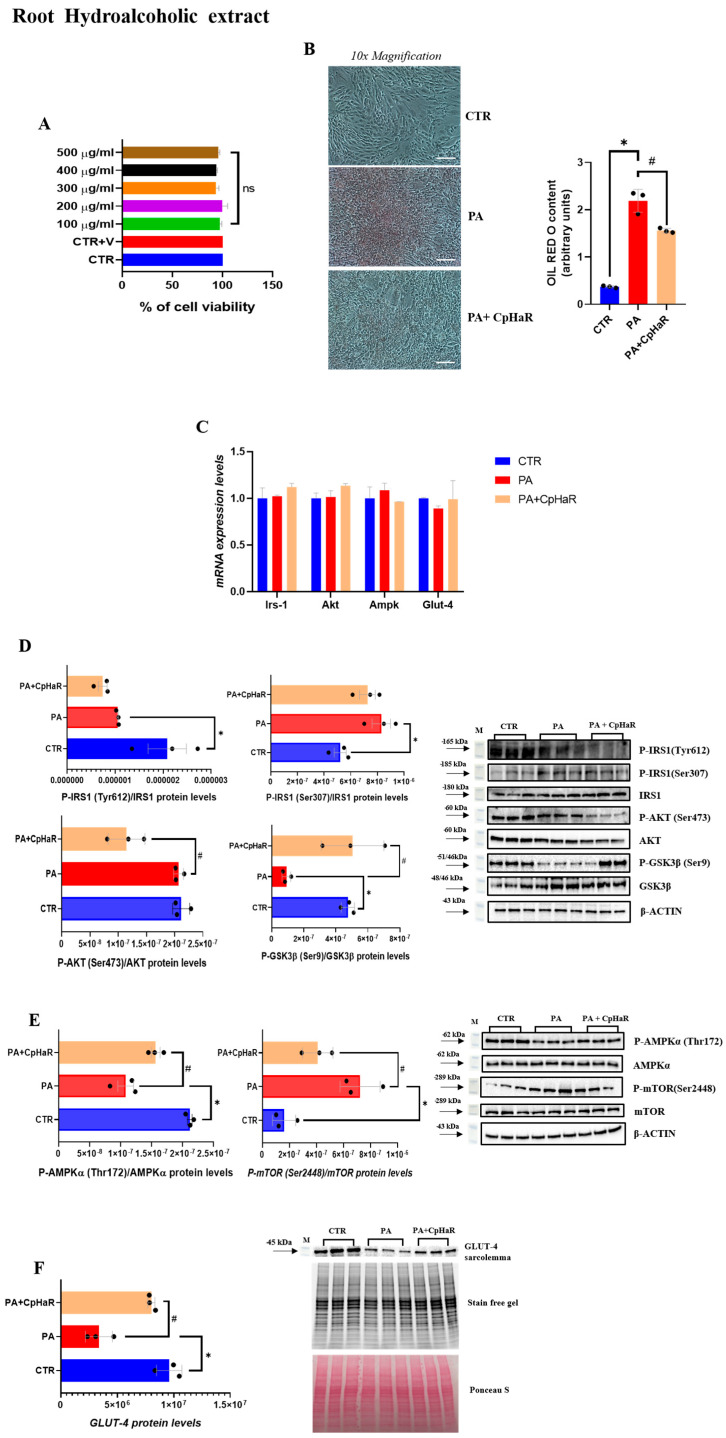
(**A**) MTT assay in L6 cells following the exposure to various concentrations of Root Hydroalcoholic extract from *C. prophetarum* L. for 24 h; (**B**) ORO staining and ORO staining quantification normalized for protein content of L6 cells treated with PA (0.75 mM) and Root Hydroalcoholic extract from *C. prophetarum* L. (300 µg/mL). Cells were visualized under a 10× magnification, with a scale bar of 2 μm; (**C**) mRNA expression levels of Irs-1, Akt, Ampk, Glut-4. The mRNA level was normalized to that of β-actin and GAPDH; (**D**,**E**) Representative immunoblots of P-IRS1 (Tyr612), P-IRS1 (Ser307), P-AKT (Ser473), P-GSK3β (Ser9), P-AMPKα (Thr172), and P-mTOR (Ser2448). Histograms represent the results of the densitometric analysis of the immunoblots, normalized to the corresponding total (non-phosphorylated) form of each protein and to β-actin as a loading control; (**F**) Representative immunoblots of GLUT4 detected in the plasma membrane fraction. Histograms represent the results of the densitometric analysis of the immunoblots. Stain-Free technology was used as normalization. Results are expressed as mean ± SEM (*n* = 3/group). One-way ANOVA was used for statistical analysis. * *p* < 0.05 vs. CTR; # *p* < 0.05 vs. PA. **CTR:** control; **PA:** palmitic acid; **PA + CpHaR:** palmitic acid plus root hydroalcoholic extract.

Similarly, treatment of L6 myotubes with the root hydroalcoholic extract of *C. prophetarum* (100–500 µg/mL, 24 h) did not alter cell viability (Figure 4A). Consistent with the observations made for the stem extract, ORO staining revealed a reduction in lipid accumulation in the PA + CpHaR group compared to the PA-treated cells (Figure 4B). No significant changes in total Irs-1, Akt, Ampk, and Glut-4 expression were observed among the experimental groups (Figure 4C). Regarding the phosphorylation levels of proteins involved in the insulin signaling pathway, in L6-myotubes treated with PA + CpHaR, we observed that the phosphorylation of IRS-1 at Tyr612 was not increased compared to PA-treated myotubes, whereas phosphorylation of IRS-1 at Ser307 was reduced by approximately 12%, although this decrease did not reach statistical significance (Figure 4D). Furthermore, we found that AKT phosphorylation was markedly decreased in the PA + CpHaR group compared to PA-treated myotubes, while GSK-3β phosphorylation at Ser9 was significantly increased compared to the PA group, restoring phosphorylation levels to those observed in the control myotubes (Figure 4D). A significant increase in AMPKα phosphorylation levels was observed in the PA + CpHaR group compared to PA-treated myotubes, whereas treatment with PA + CpHaR resulted in a marked decrease in mTOR phosphorylation (Figure 4E). Moreover, in myotubes treated with PA + CpHaR, GLUT4 levels at the plasma membrane were significantly higher than in PA-treated cells (Figure 4F).

### 2.5. Leaf Hydroalcoholic Extract of C. prophetarum Fails to Ameliorate Insulin Signaling in Insulin-Resistant L6 Myotubes

**Figure 5:**
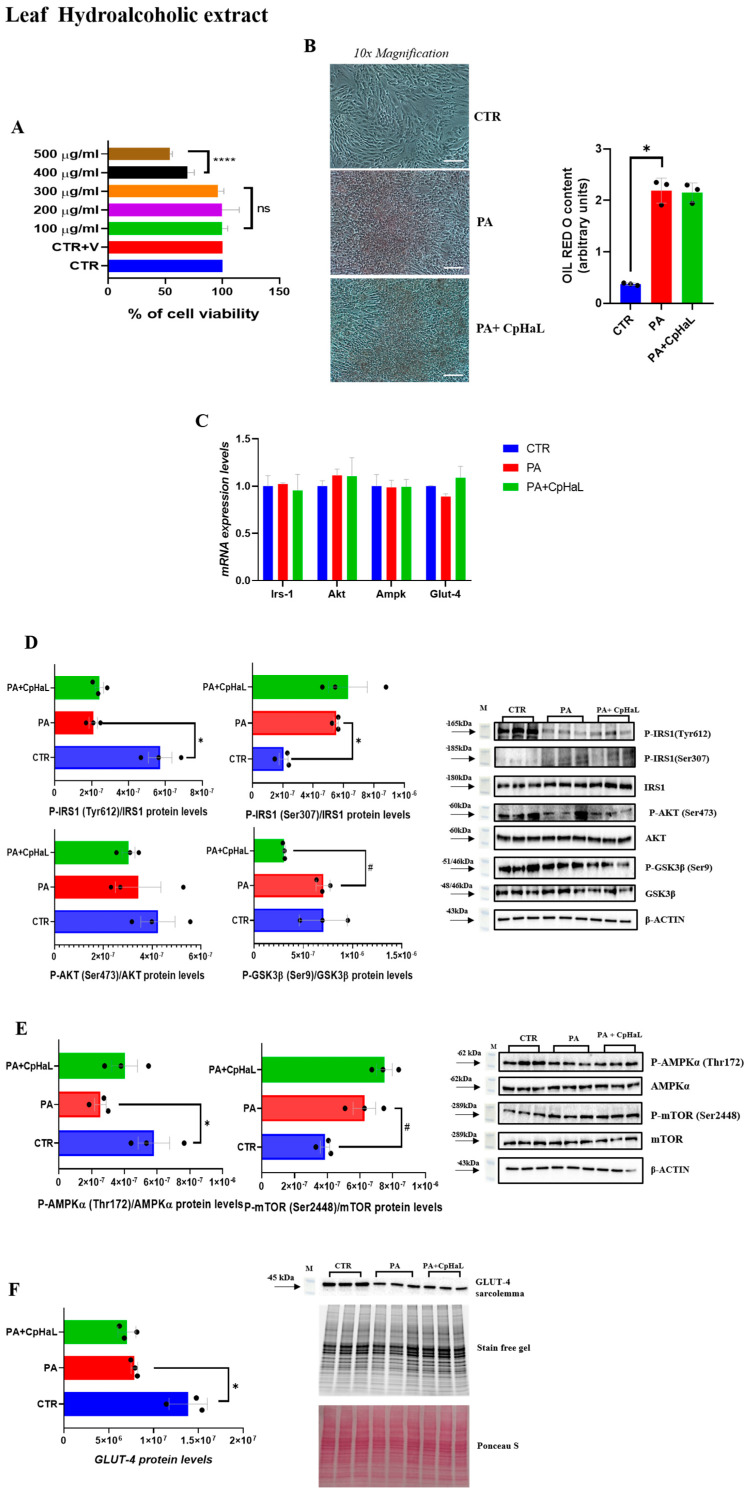
(**A**) MTT assay in L6 cells following the exposure to various concentrations of Leaf Hydroalcoholic extract from *C. prophetarum* L. for 24 h; (**B**) ORO staining and ORO staining quantification normalized for protein content of L6 cells treated with PA (0.75 mM) and Leaf Hydroalcoholic extract from *C. prophetarum* L. (300 µg/mL). Cells were visualized under a 10× magnification, with a scale bar of 2 μm; (**C**) mRNA expression levels of Irs-1, Akt, Ampk, Glut-4. The mRNA level was normalized to that of B-actin and GAPDH; (**D**,**E**) Representative immunoblots of P-IRS1 (Tyr612), P-IRS1 (Ser307), P-AKT (Ser473), P-GSK3β (Ser9), P-AMPKα (Thr172), and P-mTOR (Ser2448). Histograms represent the results of the densitometric analysis of the immunoblots, normalized to the corresponding total (non-phosphorylated) form of each protein and to β-actin as a loading control; (**F**) Representative immunoblots of GLUT4 detected in the plasma membrane fraction. Histograms represent the results of the densitometric analysis of the immunoblots. Stain-Free technology was used as normalization. Results are expressed as mean ± SEM (*n* = 3/group). One-way ANOVA was used for statistical analysis. * *p* < 0.05 vs. CTR; **** *p* < 0.0001 vs. CTR; # *p* < 0.05 vs. PA. **CTR:** control; **PA:** palmitic acid; **PA + CpHaL:** palmitic acid plus leaf hydroalcoholic extract.

Treatment of L6 myotubes with the leaf hydroalcoholic extract of *C. prophetarum* at concentrations between 100 and 300 µg/mL did not significantly affect cell viability (Figure 5A). In contrast, exposure to higher concentrations (400 and 500 µg/mL) for 24 h resulted in a significant reduction in cell viability, indicating cytotoxic effects (Figure 5A). Subsequent analyses by ORO staining revealed that treatment with PA + CpHaL did not reduce lipid accumulation compared to the PA group (Figure 5B). In parallel, Irs-1, Akt, Ampk, and Glut-4 expression levels were evaluated and, as expected, remained unchanged among the experimental groups (Figure 5C). Furthermore, the analysis of the phosphorylation levels of key proteins involved in the insulin signaling pathway revealed that, in PA + CpHaL–treated myotubes, IRS-1 phosphorylation at Tyr612 was not increased compared to PA-treated myotubes, while phosphorylation at Ser307 was not reduced after the treatment with the leaf hydroalcoholic extract (Figure 5D). Likewise, GSK-3β phosphorylation was markedly decreased compared to PA group, and no significant changes were detected in AKT phosphorylation levels (Figure 5D). In addition, mTOR phosphorylation was not reduced in the PA + CpHaL group compared to the PA-treated cells (Figure 5E). Additionally, no detectable change in GLUT4 translocation was observed in the PA + CpHaL group compared with PA-treated myotubes (Figure 5F).

## 3. Discussion

T2DM, pathophysiologically characterized by peripheral insulin resistance and chronic hyperglycemia, represents one of the most rapidly escalating global health issues of the 21st century [39]. Despite the availability of several pharmacological interventions, achieving and maintaining long-term glycemic control in patients with T2DM remains a major therapeutic challenge, underscoring the urgent need for novel and more effective treatment strategies [40,41]. Insulin resistance itself represents a multifaceted metabolic dysfunction involving numerous molecular and physiological pathways [42,43]. The use of plant-derived extracts has gained increasing attention as a promising alternative or complementary therapeutic strategy to conventional pharmacological approaches, offering potential benefits for the treatment of insulin resistance [44]. In this context, the present study investigated the potential of the stem, root and leaf hydroalcoholic extracts from *C. prophetarum* to mitigate fatty acid–induced insulin resistance. Firstly, to evaluate their effects, the cytotoxic profile of the extracts was assessed on L6 myotubes. The hydroalcoholic extracts from *C. prophetarum* showed no significant cytotoxic effects on L6 myotubes across a broad range of concentrations. However, the leaf extract at concentrations of 400 and 500 µg/mL markedly reduced cell viability by approximately 50–70%, suggesting a dose-dependent cytotoxic effect.

Phosphorylation of IRS-1 at specific amino acid residues plays a critical role in regulating insulin signaling across various tissues [45]. While tyrosine phosphorylation of IRS-1 promotes insulin action, serine phosphorylation can inhibit insulin signaling, thereby contributing to the development of insulin resistance [46,47].

This impairment is commonly associated with alterations in IRS-1/PI3K/Akt pathway, which becomes dysregulated under insulin-resistant conditions [48,49,50]. Our data demonstrated that treatment with palmitic acid induced a marked accumulation of intracellular lipids in L6 myotubes. This increase in lipid deposition was concomitant with a reduction in the phosphorylation levels of key proteins involved in the insulin signaling pathway. Exposure of insulin-resistant L6 myotubes to the stem hydroalcoholic extract resulted in a significant increase in the phosphorylation levels of IRS-1 (Tyr612), Akt, and GSK3β compared to the PA-treated group. Conversely, IRS1 phosphorylation at Ser307 was significantly reduced in the PA + CpHaS group compared to myotubes treated with PA alone. Overall, these findings indicate that the stem hydroalcoholic extract effectively counteracts palmitic acid–induced insulin resistance in L6 myotubes by restoring the phosphorylation of key components of the insulin signaling pathway. Alterations in the IRS-1/PI3K/Akt pathway under insulin-resistant conditions lead to defective GLUT4 trafficking to the plasma membrane, thereby limiting glucose uptake [51,52]. In line with these observations, PA-treated L6 myotubes exhibited a marked reduction in GLUT4 localization at the plasma membrane, confirming the establishment of an insulin-resistant phenotype in our model. Notably, treatment with the stem and root hydroalcoholic extract significantly restored GLUT4 translocation to the plasma membrane compared with PA-treated myotubes. These findings demonstrate that the stem and root hydroalcoholic extract can restore insulin signaling and effectively rescue GLUT4 translocation to the plasma membrane in L6 myotubes. In contrast, treatment with the root extract did not induce significant changes in the phosphorylation levels of the main proteins involved in the insulin signaling pathway, except for GSK3β, whose phosphorylation was increased in the PA + CpHaR group compared to PA-treated myotubes. These findings may be attributed to bioactive compounds within the extracts known to influence insulin signaling. Apigenin and related flavones, for instance, have demonstrated antidiabetic properties in previous studies [53]. Supporting this, our phytochemical analysis identified glycosylated flavones such as apigenin-*C*,*O*-dihexoside (the most abundant compound in the stem and leaf extracts) and apigenin-6-*C*-hexoside (also called Isovitexin) (Supplementary Figures S1 and S2), with higher concentrations in the stem extract (Figure 2, Table 1) which was reported for its promising anti-diabetic potential by inhibiting Rat Lens Aldose Reductase (RLAR), Human Recombinant Aldose Reductase (HRAR), advanced glycation endproducts (AGEs) formation and Protein Tyrosine Phosphatase 1B (PTP1B) [54]. As it has been observed in Figure 3D (for the stem) and 5D (for the leaf) the difference in the level of Akt between the leaf and stem could be due to the existence of more diversified secondary metabolites in the stem that could have synergistic effect against insulin resistance. In addition, apigenin-6-*C*-hexoside (also called Isovitexin) is mainly identified in the stem hydroalcoholic extract with different forms which might contribute to the difference in the bioactivity of stem and leaf hydroalcoholic extracts (Table 1). As observed in the cell viability result, the leaf decoction showed a relatively lower cell viability than the stem counterpart which might have contributed to the observed differences. Moreover, cucurbitacins, a class of triterpenoids predominantly found in the root extract (Supplementary Figure S3), and characteristic of the Cucurbitaceae family, have also been reported to exert antidiabetic effects through different mechanisms [55]. Another structurally related flavone, chrysin, has been reported to regulate glucose and lipid metabolism by modulating the AMPK/PI3K/Akt pathway in insulin-resistant HepG2 cells and in C57BL/6J mice with T2DM induced by a high-fat diet and streptozotocin (HFD/STZ model) [56]. Hypoglycemic effects have also been reported for other flavonoids, such as apigenin-6-*C*-β-fucopyranoside and apigenin-6-*C*-(2″-*O*-α-rhamnopyranosyl)-β-fucopyranoside, isolated from *Averrhoa carambola* leaf in hyperglycemic rats [57]. In general, flavonoids are known to stimulate the PI3K/Akt pathway, leading to a downregulation of key gluconeogenic enzymes such as phosphoenolpyruvate carboxykinase and glucose-6-phosphatase, thereby reducing gluconeogenesis and enhancing glycogen synthesis [58].

Beyond their direct effects on glucose metabolism, flavonoids are also recognized for their anti-inflammatory, lipid-lowering, and antioxidant properties, which contribute significantly to the mitigation of insulin resistance and the management of metabolic disorders [59]. Supporting this, a study on kaempferol, a flavonoid isolated from *Ginkgo biloba*, demonstrated enhanced AKT and hexokinase activity, thereby improving whole-body insulin sensitivity in HFD obese mice [60].

The activation of AMPK has been shown to increase insulin sensitivity by preserving mitochondrial function and preventing the accumulation of fatty acids in liver and skeletal muscle [61,62,63]. Furthermore, AMPK activation contributes to improved insulin responsiveness by promoting GLUT4 translocation and inhibiting the mTOR signaling pathway [64,65,66,67]. In our study, treatment with stem and root hydroalcoholic extracts from *C. prophetarum* L. resulted in a significant increase in AMPKα phosphorylation levels and a concomitant reduction in mTOR phosphorylation compared with the PA-treated group, suggesting that both extracts may enhance insulin signaling through modulation of the AMPK–mTOR pathway. While the stem extract further improves insulin signaling by activating the IRS-1/PI3K/Akt pathway in addition to modulating AMPK–mTOR, the root extract primarily enhances insulin sensitivity via the AMPK–mTOR pathway. Consistently, in myotubes treated with PA + CpHaR, GLUT4 translocation to the plasma membrane was significantly increased compared with PA-treated myotubes. This suggests that the activation of AMPK–mTOR is a key mechanism underlying the insulin-sensitizing effects of the root extract, which may be attributed to cucurbitacins, bioactive compounds commonly found in the Cucurbitaceae family [68].

Several plant-derived metabolites have been reported to alleviate insulin resistance by modulating the AMPK/mTOR/p70S6K pathways [69,70,71]. In 3T3-L1 cells, apigenin was shown to activate AMPK in a dose-dependent manner, suggesting a key role in its antidiabetic action [72]. Similarly, genistein enhanced glucose uptake in L6 myotubes under both normal and high glucose conditions, through mechanisms involving the PI3K, mTOR, and AMPK pathways [73]. The decoction extract of *C. prophetarum* demonstrated insulin-sensitizing effects by modulating protein phosphorylation in the IRS-1/PI3K/AKT, AMPK, and mTOR/p70S6K pathways, likely due to its high content of flavonoids and related bioactive compounds [15]. The activation of AMPK leads to the phosphorylation of downstream targets such as Acetyl-CoA carboxylase (ACC) at Ser79, an inhibitory site that blocks the conversion of Acetyl-CoA to malonyl-CoA. This internal process promotes mitochondrial fatty acid oxidation by facilitating the entry of long-chain fatty acids into mitochondria and reducing intracellular lipid accumulation [74]. Consistent with these observations, the treatment of L6 myotubes with hydroalcoholic extracts of stem and root led to a marked reduction in lipid accumulation compared to PA-treated myotubes (Figure 3B and Figure 4B).

The use of aqueous and hydroalcoholic solvents has been shown to yield extracts with a different chemical composition [16]. In our study, hydroalcoholic extraction from the solvent matrix resulted in distinct chemical profiles which were reflected in a differential modulation of protein phosphorylation of the insulin signaling. The hydroalcoholic leaf and stem extracts uniquely contained regaloside A/F (**2**, **4**) and luteolin *C*,*O*-dihexoside (**3**), while apigenin malonylhexoside was exclusive to these extracts, despite apigenin *C*-hexoside being common to both. In contrast, the root decoction extract contained raffinose, fructoselysine, protocatechuyl pentoside, and cucumegastigmane I/II, whereas the root hydroalcoholic extract was rich in cucurbitacins (**10**, **11**, **13**, **14**) and polyunsaturated fatty acids (PUFAs; **12**, **15**), which are typical of the Cucurbitaceae family.

In conclusion, this study demonstrates that hydroalcoholic extracts from the stem of *C. prophetarum* can effectively alleviate PA-induced insulin resistance in L6 skeletal muscle cells, whereas the root extract shows only partial effects, which appear to be mediated through the coordinated modulation of the IRS-1/PI3K/AKT and AMPK/mTOR pathways. Notably, the stem hydroalcoholic extract elicited the most pronounced effects, significantly enhancing insulin signaling. Equally important, this study emphasizes the critical role of comprehensive chemical profiling in understanding the bioactivity of plant-based extracts. Through advanced UHPLC-QqTOF-MS/MS, we identified specific flavonoids, such as apigenin *C*,*O*-dihexoside and luteolin *C*,*O*-dihexoside, primarily in the leaf and stem, while cucurbitacins and polyunsaturated fatty acids were dominant in the root. These findings provide a molecular basis for the differential effects observed across plant organs. Furthermore, the current study builds upon previous findings on the decoctions of *C. prophetarum*, enabling a direct comparison between solvent extraction systems. The hydroalcoholic extract not only enriched the metabolite diversity but also enhanced the biological effect, revealing compounds absent in the aqueous fraction, such as apigenin malonylhexoside and multiple cucurbitacin variants. This comparison underscores the importance of solvent selection in maximizing the therapeutic potential of plant materials.

Moreover, considering these promising in vitro findings, future studies will aim to translate the insulin-sensitizing effects of stem and root hydroalcoholic extracts of *C. prophetarum* L. into in vivo models, focusing on metabolically active tissues such as skeletal muscle and adipose tissue. These studies will allow validation of the modulation of key signaling pathways, including IRS-1/PI3K/Akt and AMPK/mTOR, as well as the restoration of GLUT4 translocation, providing essential preclinical evidence for the therapeutic potential of these plant-derived extracts in the management of insulin resistance and type 2 diabetes.

## 4. Materials and Methods

### 4.1. Hydroalcoholic Plant Extract Preparation

To prepare the hydroalcoholic crude extracts, plant materials of *Cucumis prophetarum*, including leaf, stems, and roots, were collected from the area around Lake Tana region of the Abay River basin in Northwestern Ethiopia. The voucher specimen recorded as ZM001 was deposited at the National Herbarium of Addis Ababa University. The harvested plant parts were thoroughly washed under running tap water to remove surface debris, then air-dried in the shade to retain their phytochemical properties. Once dried, the materials were ground into a coarse powder. For extraction, 250 g of powdered material from each plant part was immersed in a hydroalcoholic solvent composed of methanol and water (4:1, *v*/*v*) in Erlenmeyer flasks. The plant material-to-solvent ratio was maintained at 1:6 (g/L). Maceration was performed at room temperature for 72 h with occasional stirring. After the extraction period, the mixtures were filtered, and the filtrates were concentrated using a rotary evaporator (Heidolph Hei-VAP Advantage, Schwabach, Germany) under reduced pressure, ensuring the temperature did not exceed 40 °C to protect heat-sensitive compounds. The concentrated extracts were then frozen and lyophilized using a Biocool FD-1A-50 freeze dryer (Beijing, China) to eliminate residual moisture. The final dried extracts were stored at −4 °C until further use.

### 4.2. Spectroscopic Prediction of the Hydroalcoholic Extract of C. prophetarum

The UV-Vis absorption spectra of the hydroalcoholic extracts were recorded over a wavelength range of 200–800 nm using a Mobi™ Atomic Absorption Microplate Spectrophotometer (MSE Supplies, Changwon, Republic of Korea). To identify the functional groups present in the extracts, Fourier-transform infrared (FT-IR) spectroscopy was performed using an IRXross FT-IR spectrophotometer (Shimadzu, Milan, Italy). Spectra were collected in the wavenumber range of 500–4000 cm^−1^ with a resolution of 4 cm^−1^ over 45 scans. The ATR-FT-IR spectra were acquired and further analyzed using LabSolutions IR software (v.1.60, Shimadzu, Milan, Italy).

### 4.3. UHPLC-ESI-QqTOF-MS/MS-Based Metabolic Profiling of Hydroalcoholic Extracts of C. prophetarum Leaf, Stem and Root

Qualitative metabolite profiling of the hydroalcoholic extracts from *C. prophetarum* organs was conducted using a high-resolution chromatographic and mass spectrometric approach, with minor modifications from the protocol described by Mekonnen et al. [15]. The analysis was carried out on a Shimadzu NEXERA UHPLC system (Shimadzu, Kyoto, Japan), equipped with a Luna^®^ Omega C18 column (1.6 µm; 50 × 2.1 mm i.d., Phenomenex, Torrance, CA, USA). Chromatographic separation was performed under a gradient elution mode using a binary solvent system: solvent A (water with 0.1% formic acid) and solvent B (acetonitrile with 0.1% formic acid). The gradient initiated with 5% B, ramping to 17.5% within 5 min, and then increasing to 45% at 8 min. This condition was held for 2 min before further rising to 95% B. After 1 min at 95%, the system returned to the initial condition for column re-equilibration. The flow rate was maintained at 0.5 mL/min throughout the run. For mass spectrometric analysis, a hybrid quadrupole time-of-flight (Q-TOF) instrument, AB Sciex TripleTOF^®^ 4600 (AB Sciex, Framingham, MA, USA), was employed in negative electrospray ionization (ESI) mode. Data acquisition included a full-scan TOF mode covering a mass range of 220–1000 Da, along with eight Information Dependent Acquisition (IDA) MS/MS scans in the 80–950 Da range. Source parameters were set as follows: curtain gas at 35 psi, nebulizer gas at 60 psi, heated gas at 60 psi, and ion spray voltage at −4.5 kV. The interface temperature was maintained at 600 °C, with a declustering potential of −70 V. Collision energy was set to −40 ± 5 V. Data acquisition and processing were performed using Analyst^®^ TF 1.7 and PeakView^®^ 2.2 software, respectively.

### 4.4. Cell Viability Test

L6 cells were seeded (100 μL/well) in triplicate in 96-well plates at a density of 1.5 × 10^4^ cells/well with DMEM supplemented with 10% Fetal Bovine Serum (FBS) and antibiotics (100 U/mL penicillin, and 100 U/mL streptomycin) incubated for 24 h at 37 °C in a 5% CO_2_ atmosphere [75]. Once the L6 myoblasts grew to 70–80% confluence, effective differentiation into myotubes was maintained via incubation of the cells for another 24 h with new DMEM containing 2% FBS and antibiotics (100 U/mL penicillin and 100 U/mL streptomycin) [75]. Then, L6 myotubes were incubated with different concentrations (100 μg/mL–500 μg/mL) of hydroalcoholic extract in triplicate for 24 h [76]. The myotubes were then treated with 150 µL 3-(4,5-dimethylthiazol-2-yl)-2,5-diphenyl tetrazolium bromide (MTT; 0.5 mg/mL) (Merck, Milan, Italy), and incubated for 4 h at 37 °C. Then, the supernatant was removed, and 100 µL of isopropanol was added to dissolve the produced formazan dye. Finally, the absorbance of each sample was measured using a Sinergy H1 microplate reader (BioTek, Shoreline, WA, USA) at 570 nm wavelength, and percent cell viability was calculated [77,78,79].

### 4.5. Cell Culture and Treatment

The L6 myoblast cells were cultured in *Petri* dishes and maintained in Dulbecco’s Modified Eagle’s Medium (DMEM) supplemented with 10% Fetal Bovine Serum (FBS) and antibiotics (100 U/mL penicillin and 100 U/mL streptomycin) in an incubator at 37 °C under a humidified atmosphere with 5% CO_2_. After the myoblast growth reached confluence, the differentiation of L6 cells into myotubes was performed by changing the medium with 2% FBS-supplemented DMEM and antibiotics (100 U/mL penicillin and 100 U/mL streptomycin) and incubating with refreshment of the medium. Once the L6 myoblast cells had fully differentiated, myotubes were exposed to Palmitic acid (PA) (Merck, Milan, Italy) at a concentration of 750 μM to induce a condition of insulin resistance [48,80,81,82]. After 18 h, the medium was replaced with fresh media containing PA and the hydroalcoholic extract of *C. prophetarum* (leaf, stem, or root) at 300 μg/mL for an additional 6 h. A concentration of 300 µg/mL was selected for subsequent evaluations. This concentration represented the highest dose at which none of the hydroalcoholic extracts affected cell viability. At the end of the treatment, cells were scraped, washed twice with phosphate-buffered saline (PBS), and stored at −80 °C until further use.

### 4.6. Western Blot Analysis

Cell culture pellets were homogenized in RIPA buffer. PhosSTOP™ (Roche) was added to prevent dephosphorylation of phosphorylation-sensitive proteins during sample preparation. The homogenates were first centrifuged at 14.000× *g* for 15 min at 4 °C to obtain the total lysate (Beckman Coulter S.p.A., Milan, Italy). Subsequently, an aliquot of the resulting supernatant from each sample was ultracentrifuged at 100.000× *g* for 1 h at 4 °C using a Beckman Optima TLX ultracentrifuge (Beckman Coulter S.p.A., Milan, Italy). The pellet obtained, representing the membrane-enriched fraction, was resuspended in RIPA buffer. Protein concentrations in both whole-cell lysates and membrane fractions were quantified with the DC Protein Assay (Bio-Rad Laboratories S.r.l., Segrate, Italy) [83,84,85]. Electrophoresis on SDS-PAGE gels and Western immunoblot analysis were carried out as described previously by Petito et al. [86] and Senese et al. [87] with minor modifications. Briefly, after determining the protein concentration using Bio-Rad’s DC Protein Assay, 15 µg of protein from total and membrane fraction lysates were loaded per lane, separated by SDS-PAGE, and transferred onto a nitrocellulose membrane. Precision Plus Protein™ All Blue Prestained Protein Standards (1610373; Biorad) (Molecular weight 10 to 250 KDa) were used as a protein ladder for the molecular weight reference. β-actin was used as an internal load control. The membranes were incubated with primary antibodies diluted in TBS containing 0.01% (*v*/*v*) Tween 20 (TBS-T) and 5% (*w*/*v*) bovine serum albumin (BSA), while secondary antibodies were diluted in TBS-T with 5% (*w*/*v*) nonfat dry milk. The membranes were incubated with anti-P-IRS1 (Tyr612) (Invitrogen, Carlsbad, CA, USA), anti-P-IRS1 (Ser307) (Millipore, Burlington, MA, USA), anti-IRS1 (Cell Signaling, Danvers, MA, USA), anti-P-AKT (Ser473) (Cell Signaling), anti-AKT (Cell Signaling), anti-P-GSK3β (Ser9) (ABclonal, Woburn, MA, USA), anti-GSK3β (ABclonal), anti-P-AMPKα (Thr172) (Cell Signaling), anti-AMPKα (Cell Signaling), anti-P-mTOR (Ser2448) (Cell Signaling), anti-mTOR (Cell Signaling), anti-GLUT-4 (Cell Signaling) and anti-β-Actin (Bioss) antibodies at 4 °C overnight. As secondary antibodies, peroxidase anti-rabbit IgG (abcam-1:4000 dilution) and peroxidase anti-mouse IgG (abcam-1:4000 dilution) were used. Chemiluminescent blots were imaged using the ChemiDoc XRS+ (Biorad) and analyzed using Image Lab Software 6.0.1. For GLUT-4 detection, Stain-Free technology was employed for normalization. TGX Stain-Free gels were activated for 1 min following SDS-PAGE and imaged using the ChemiDoc MP system (Bio-Rad, Hercules, CA, USA) with ImageLab software (version 6.0.1, Bio-Rad). Both Stain-Free images (post-transfer) and fluorescent blot images were acquired using the same system and software. Analysis involved quantifying total Stain-Free fluorescence and the GLUT-4 signal intensity in each lane using the ‘Lane and Bands’ tool in ImageLab. GLUT-4 signals were automatically normalized to total lane volumes provided by Stain-Free imaging within ImageLab.

### 4.7. Oil Red O (ORO) Staining of Cells

The lipid accumulation was evaluated using Oil Red O staining as described in the previous study [15]. After 6 h of exposure to hydroalcoholic extracts of *C. prophetarum* (leaf, stem, and root) at 300 μg/mL, the medium was removed, and the cells were washed with PBS 1x and fixed in 10% formalin for 1 h, followed by three washes with PBS. After treating the cells with 60% isopropanol for 5 min, they were stained with ORO working solution for 10 min at room temperature, followed by washing with 1X PBS to remove any unbound stain. Images were acquired using a Nikon Eclipse TE300 optical microscope (Nikon, New York, NY, USA) at 10× magnification. To quantify the Oil Red O content, 100% isopropanol was added to each sample, shaken for 5 min, and the absorbance was measured at 500 nm using Synergy H1 microplate reader (BioTek, Shoreline, WA, USA). The quantification of ORO staining was then normalized to the total protein content, ensuring that differences in cell number or protein concentration did not confound the assessment of lipid accumulation and allowing for an accurate comparison of lipid levels across all experimental conditions [88,89].

### 4.8. RNA Isolation and Quantitative Real-Time PCR (qRT-PCR) Analysis

Total RNA was isolated from cells using the miRNeasy Mini Kit (Qiagen, Hilden, Germany) in accordance with the manufacturer’s instructions. RNA concentration and purity were assessed with a NanoDrop 2000c spectrophotometer (Thermo Scientific, Waltham, MA, USA). cDNA was synthesized from 1 μg of total RNA in a final volume of 20 μL using SuperScript II Reverse Transcriptase kit (Thermo Scientific, Waltham, MA, USA). Quantitative real-time PCR (qRT-PCR) was performed using gene-specific primers (50 nM), SensiFAST SYBR Hi-ROX mix (Meridian Bioscience, Cincinnati, OH, USA), and 2 μL of cDNA template. Following amplification, melting curve analysis was carried out from 55 °C to 95 °C to confirm amplification specificity and product homogeneity. Relative mRNA expression levels were calculated using the 2^−ΔΔCT^ method and normalized to the reference genes β-actin and GAPDH, which were stable under the experimental conditions. Primer sequences were designed using Primer3 [90] and synthesized and sequence-verified by Eurofins Genomics (Ebersberg, Germany). The primers used for PCR are listed below:

AKT forward: 5′-CCTCAGCACCTGAGTTGTCA-3′
AKT reverse: 5′-CTGTGGCTGATGGACTCAAA-3′
AMPK forward: 5′-CATCAAGCAGGACGTTTTCA-3′
AMPK reverse: 5′-TCCAGCAGATCCTTTCTGGT-3′
GLUT-4 forward: 5′-TTGCCCTTCTGTCCTGAGAG-3′
GLUT-4 reverse: 5′-CGCTTTAGACTCTTTCGGGC-3′
IRS-1 forward: 5′-GGCTGACTCCAAGAACAAGC-3′
IRS-1 reverse: 5′-CTTGTTCAGCCTCGCTATCC-3′
β-ACTIN forward: 5′-GGAGATTACTGCCCTGGCTCCTA-3′;
β-ACTIN reverse: 5′-GACTCATCGTACTCCTGCTTGCTG-3′
GAPDH forward: 5′-GCACCGTCAAGGCTGAGAAC-3′
GAPDH reverse: 5′-TGGTGAAGACGCCAGTGGA-3′

### 4.9. Ethical Consideration

The Institutional Review Board (IRB) of the College of Health Sciences, Addis Ababa University (AAU) provided an ethical clearance (protocol number: 097/22/Biochemistry). Memorandum of Understanding (MoU), Material Transfer Agreement, and shipping permit were obtained from AAU and EBI, Ethiopia. Under the above governance, the leaf, stem, and root powders of *C. prophetarum* L. were only used for the specific research collaboration.

### 4.10. Statistical Analysis

Data were analyzed with GraphPad Prism 8 (GraphPad Software, San Diego, CA, USA) and are presented as mean ± SEM. Statistical significance between groups was determined using One-Way ANOVA followed by Tukey’s post hoc test, with *p* < 0.05 considered significant.

## Figures and Tables

**Table 1:** Putatively identified compounds from hydroalcoholic extracts of *Cucumis prophetarum* leaf, stem, and root analyzed by UHPLC-ESI-QqTOF-MS.

Peak	Rt	[M−H]^−^ (*m*/*z* Found)	Error (ppm)	RDB	Molecular Formula	Tentative Assignment	CpHaL	CpHaS	CpHaR
1	2.519	401.1451	−0.6	6	C_18_H_26_O_10_	benzyl pentosyl hexoside	3.0	6.9	-
2	3.227	399.1292	−1.2	7	C_18_H_24_O_10_	regaloside A	5.4	4.5	-
3	3.475	609.1454	−1.2	13	C_27_H_30_O_16_	luteolin-*C*,*O*-dihexoside	1.3	0.7	-
4	3.729	429.1403	0.2	7	C_19_H_26_O_11_	regaloside F	1.4	1.1	-
5	4.113	593.1515	0.5	13	C_27_H_30_O_15_	apigenin-*C*,*O*-dihexoside	78.1	55.3	-
6	5.159	431.0985	0.3	12	C_21_H_20_O_10_	apigenin-6-C-hexoside	0.4	1.1	-
7	5.750	473.1097	1.6	13	C_23_H_22_O_11_	apigenin-6-C-acetylhexoside	0.3	4.0	-
8	5.944	473.1103	2.9	13	C_23_H_22_O_11_	apigenin-6-C-acetylhexoside	-	2.1	-
9	6.366	517.0999	2.2	14	C_24_H_22_O_13_	apigenin-6-C-malonylhexoside	-	2.1	-
10	7.796	533.3128	1.5	8	C_30_H_46_O_8_	cucurbitacin H	-	-	7.1
11	7.905	515.3006	−1.6	9	C_30_H_44_O_7_	cucurbitacin D/L	-	-	7.6
12	8.256	327.2177	0	3	C_18_H_32_O_5_	trihydroxyoctadecadienoic acid	8.3	2.2	14.3
13	8.467	517.3176	1.0	8	C_30_H_46_O_7_	cucurbitacin F	-	0.2	20.5
14	8.695	561.3072	0.5	9	C_31_H_46_O_9_	cucurbitacin A	-	0.4	15.4
15	8.714	329.2333	−0.1	2	C_18_H_34_O_5_	trihydroxyoctadecenoic acid	2.1	13.8	24.5
16	9.168	309.2066	−1.7	4	C_18_H_30_O_4_	hydroperoxyoctadecatrienoic acid	-	7.4	10.7

The color shading represents the relative abundance of each compound in the respective extract: light color indicates complete absence (0%), while deeper colors correspond to increasing concentrations. **CpHaL** 0% ■ → 100% ■ **CpHaS** 0% ■ → 100% ■ **CpHaR** 0% ■ → 100% ■.

## Data Availability

The original contributions presented in this study are included in the article and as Supplementary Materials. Further information can be provided upon request from the corresponding authors.

## Supplementary Materials

The following supporting information can be downloaded at: https://www.mdpi.com/article/10.3390/molecules31020307/s1, Figure S1. TOF-MS/MS spectra of tentatively identified Benzyl pentosyl hexoside (**1**), regaloside A (**2**), luteolin-6-C-hexoside-7-O-hexoside (**3**), regaloside F (**4**), and Apigenin-6-C-hexoside-7-O-hexoside (**5**). Figure S2. TOF-MS/MS spectra of tentatively identified apigenin-6-C-hexoside (**6**), apigenin-6-C-acetylhexoside (**7**), apigenin-6-C-acetylhexoside (**8**), apigenin-6-C-malonylhexoside (**9**). Figure S3. TOF-MS/MS spectra of tentatively identified cucurbitacin H (**10**), cucurbitacin D/L (**11**), trihydroxyoctadecadienoic acid (**12**), cucurbitacin F (**13**), cucurbitacin A (**14**), trihydroxyoctadecenoic acid (**15**), hydroperoxyoctadecatrienoic acid (**16**).

## Abbreviations

Akt	Protein kinase B
AMPK	AMP-activated protein kinase
DMEM	Dulbecco’s Modified Eagle’s Medium
FBS	Fetal bovine serum
GLUT4	Glucose Transporter 4
GAPDH	Glyceraldehyde-3-phosphate Dehydrogenase
IRS-1	Insulin receptor substrate-1
mTOR	mammalian target of rapamycin
PBS	Phosphate-buffered saline
PI3K	Phosphoinositide 3-kinase
PDK1	Phosphoinositide-dependent protein kinase-1
RIPA	Radioimmunoprecipitation assay
SDS-PAGE	Sodium dodecyl sulfate-polyacrylamide gel electrophoresis
T2DM	Type 2 diabetes mellitus
GSK3β	Glycogen synthase kinase-3β
PUFAs	Polyunsaturated fatty acids
