# Supplementary material for: Hydroalcoholic Extracts of Cucumis prophetarum L. Affect the Insulin Signaling Pathway in an In Vitro Model of Insulin-Resistant L6 Myotubes
Source: Molecules. 2026 Jan 15;31(2):307. doi: 10.3390/molecules31020307 (PMC12844345; doi:10.3390/molecules31020307)
Supplement: Supplementary file 1 [file molecules-31-00307-s001.zip › molecules-4012918-supplementary.pdf]

Supplementary Figures

1

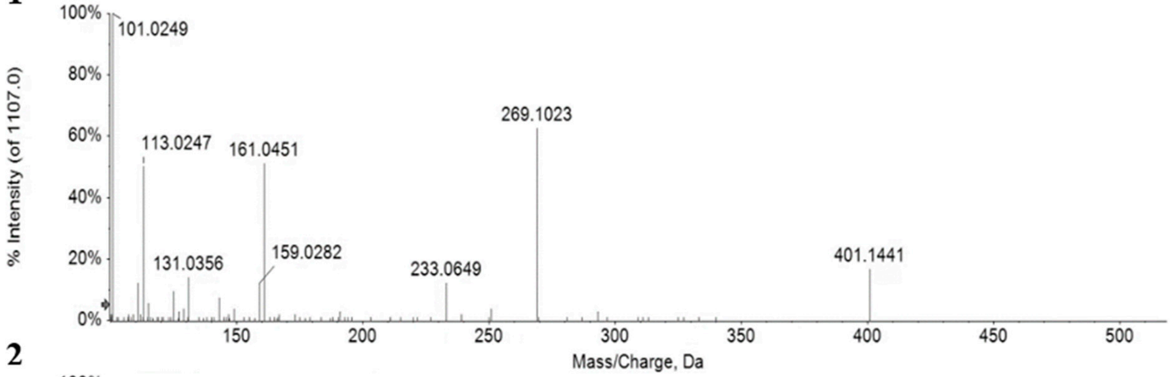

2

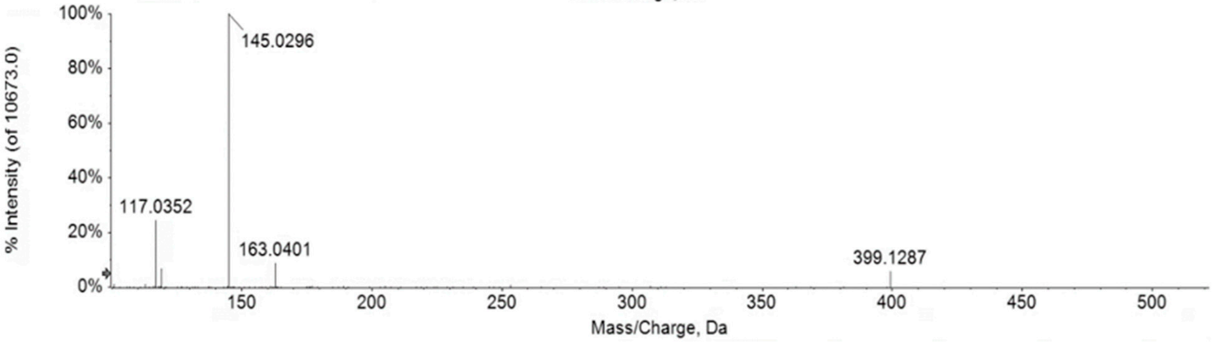

3

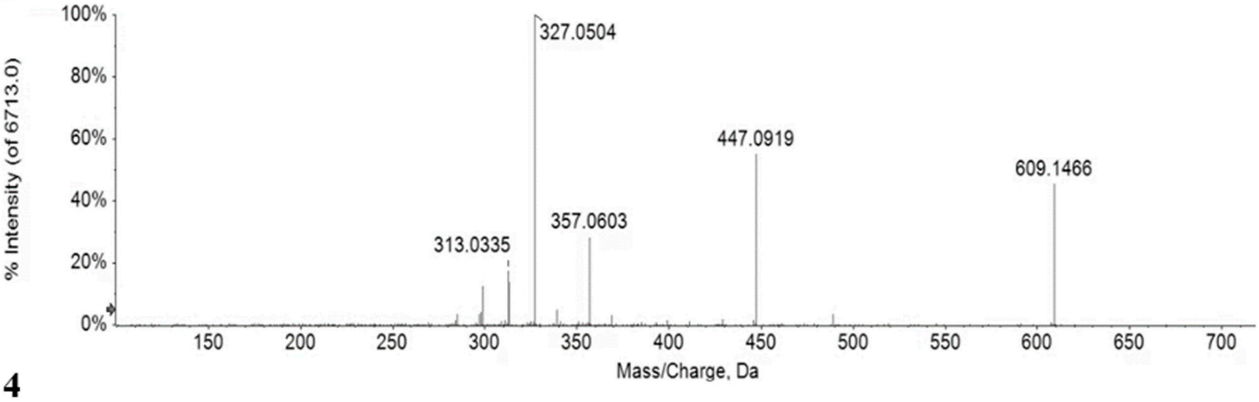

4

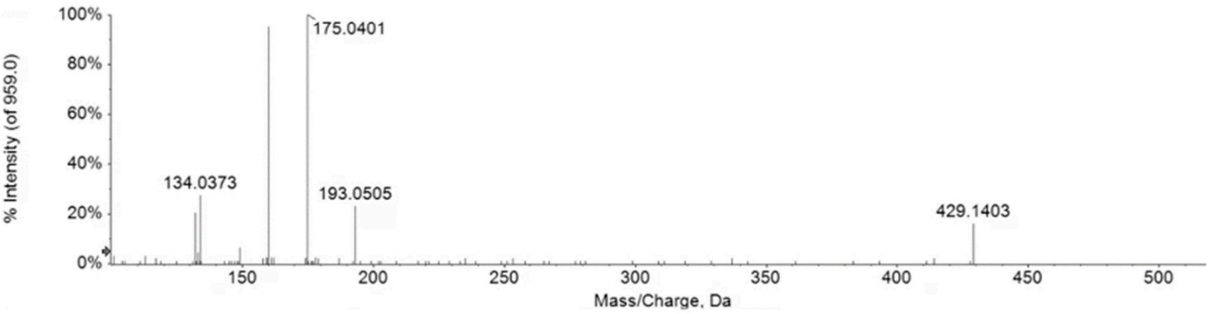

5

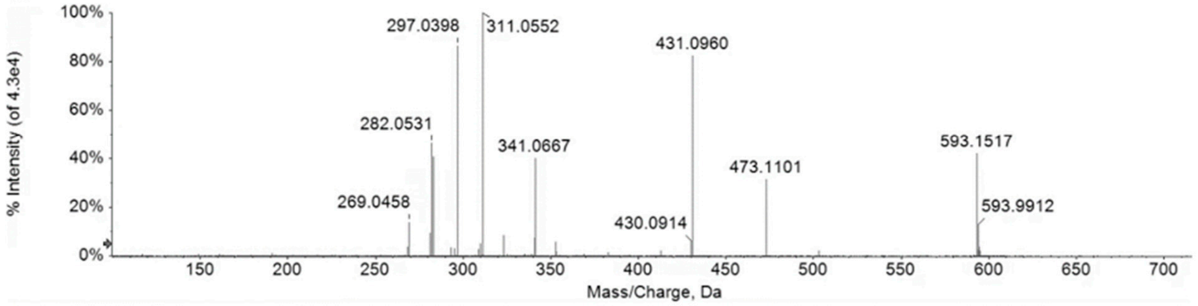

Supplementary Figure S1. TOF-MS/MS spectra of tentatively identified Benzyl pentosyl hexoside**(1),** regaloside A(**2**),luteolin-6-C-hexoside-7-O-hexoside(**3**),regaloside F(**4**), and Apigenin-6-C-hexoside-7-O-hexoside**(5)**

6

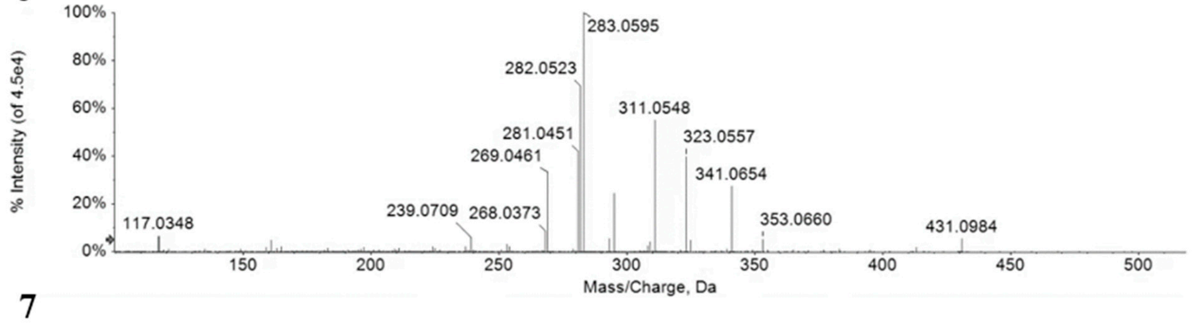

7

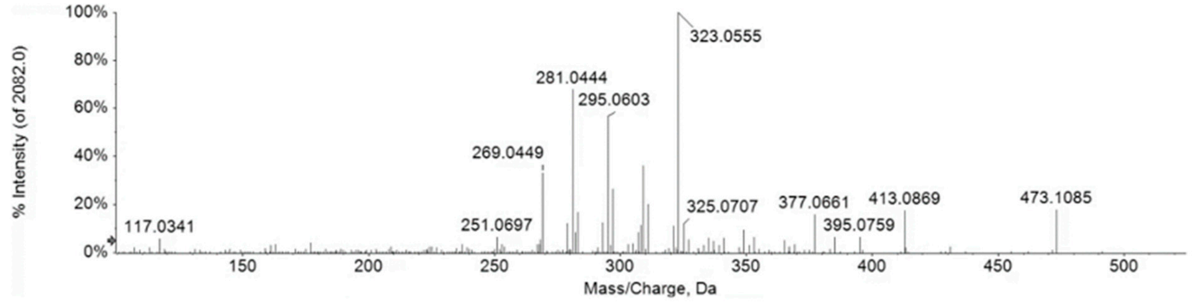

8

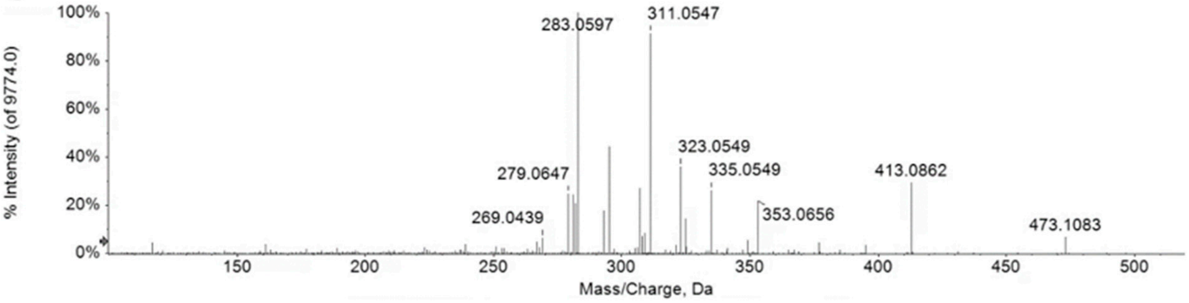

9

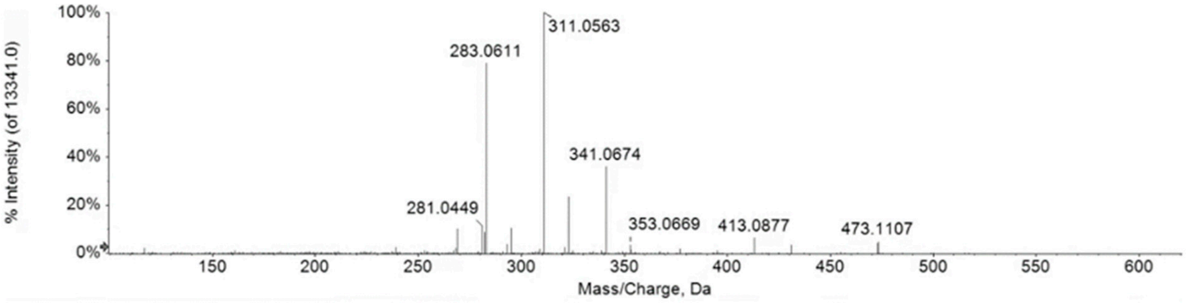

Supplementary Figure S2. TOF-MS/MS spectra of tentatively identified apigenin-6-C-hexoside(**6**),apigenin-6-C-acetylhexoside(**7**),apigenin-6-C-acetylhexoside(**8**),apigenin-6-C-malonylhexoside(**9**)

10

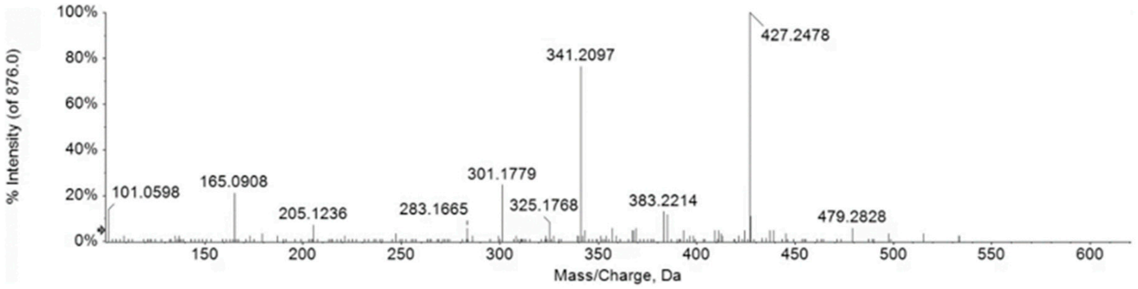
100%
80%
60%
40%
20%
0%
% Intensity (of 876.0)
427.2478
341.2097
301.1779
165.0908
101.0598
205.1236
283.1665
325.1768
383.2214
479.2828
150 200 250 300 350 400 450 500 550 600
Mass/Charge, Da

11

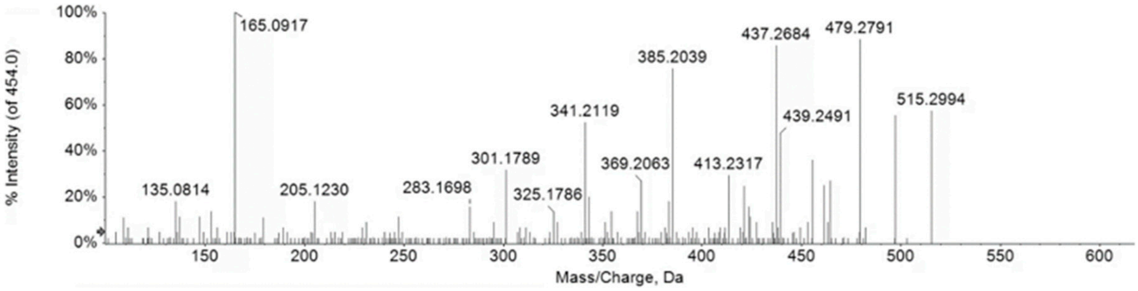
100%
80%
60%
40%
20%
0%
% Intensity (of 454.0)
165.0917
437.2684
479.2791
385.2039
515.2994
341.2119
439.2491
301.1789
369.2063
413.2317
135.0814
205.1230
283.1698
325.1786
150 200 250 300 350 400 450 500 550 600
Mass/Charge, Da

12

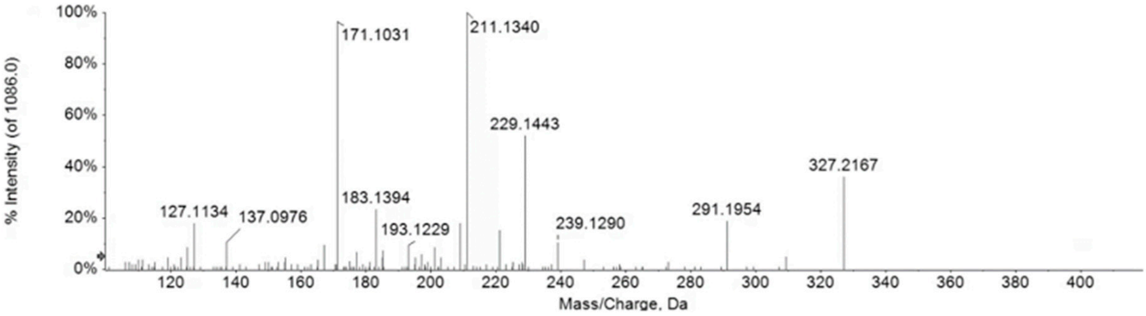
100%
80%
60%
40%
20%
0%
% Intensity (of 1086.0)
171.1031
211.1340
229.1443
327.2167
183.1394
127.1134
137.0976
193.1229
239.1290
291.1954
120 140 160 180 200 220 240 260 280 300 320 340 360 380 400
Mass/Charge, Da

13

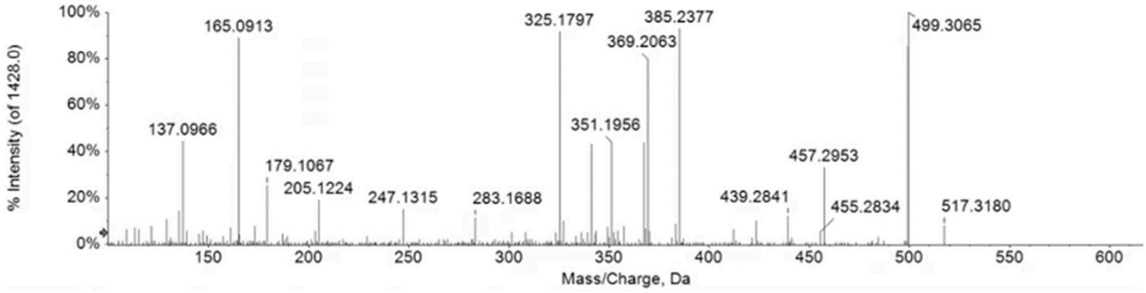
100%
80%
60%
40%
20%
0%
% Intensity (of 1428.0)
165.0913
325.1797
385.2377
369.2063
499.3065
137.0966
351.1956
457.2953
179.1067
205.1224
247.1315
283.1688
439.2841
455.2834
517.3180
150 200 250 300 350 400 450 500 550 600
Mass/Charge, Da

14

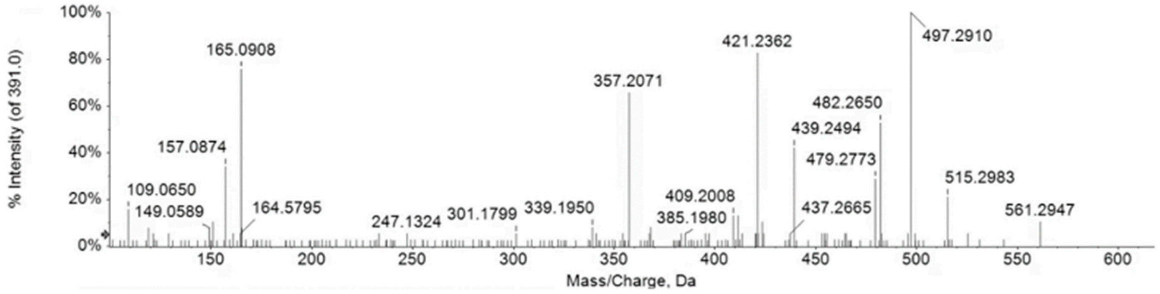

15

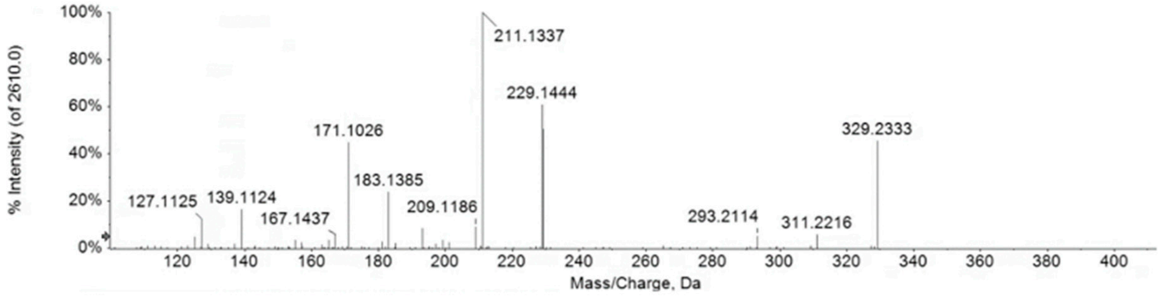

16

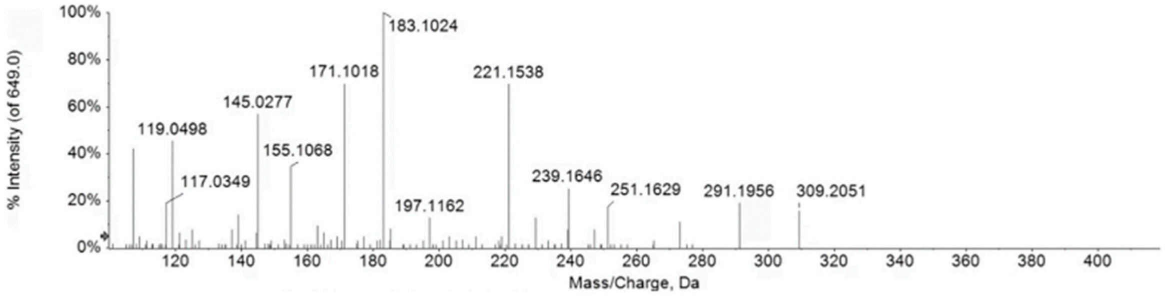

Supplementary Figure S3. TOF-MS/MS spectra of tentatively identified cucurbitacin H(**10**), cucurbitacin D/L(**11**), trihydroxyoctadecadienoic acid(**12**),cucurbitacin F(**13**), cucurbitacin A(**14**), trihydroxyoctadecenoic acid(**15**), hydroperoxyoctadecatrienoic acid(**16**)
